# Downregulation of GLYAT correlates with tumour progression and poor prognosis in hepatocellular carcinoma

**DOI:** 10.1111/jcmm.70197

**Published:** 2024-11-04

**Authors:** Fengchen Jiang, Shuiping Zhou, Chuanlong Xia, Jiale Lu, Bin Wang, Xiaowei Wang, Jiandong Shen, Wei Ding, Mengjie Yin, Feng Dai, Shouzhong Fu

**Affiliations:** ^1^ Department of Interventional Angiology Affiliated Nantong Hospital 3 of Nantong University Nantong China; ^2^ Comparative Medicine Institution, Nantong University Nantong China; ^3^ School of Medicine Nantong University Nantong China

**Keywords:** GLYAT, hepatocellular carcinoma, immunotherapy, prognosis, tumour progression

## Abstract

Glycine N‐acyltransferase (GLYAT), known to influence glycine metabolism, has been implicated in the progression of various malignant tumours. However, its clinical relevance in hepatocellular carcinoma (HCC) remains unexplored. Here, GLYAT expression levels in HCC tissues were significantly reduced compared to normal liver tissues. Similarly, GLYAT expression levels in Huh 7, HepG2, PLC and SK‐HEP1 were lower than those in LO2. Receiver operating characteristic curve analysis demonstrated that GLYAT exhibited good diagnostic performance for HCC. Kaplan–Meier analyses suggested that decreased GLYAT expression was correlated with poorer progress in HCC. Low GLYAT expression was significantly associated with gender and histologic grade. Multivariate Cox regression analysis identified low GLYAT expression and T stage as independent prognostic factors. Nomograms based on GLYAT mRNA expression and T stage showed good concordance with actual survival rates at 1, 2, 3 and 5 years. Moreover, GLYAT downregulation in the Huh 7 cell line enhanced cell proliferation, invasion and migration abilities, while GLYAT overexpression in the HepG2 cell line inhibited these abilities. HCC patients with low GLYAT expression exhibited a predisposition to immune escape and poor response to immunotherapy. This research revealed that GLYAT holds promise as both a prognostic biomarker and a potential therapeutic target in HCC.

## 1 INTRODUCTION

In 2022, primary hepatocellular carcinoma (PHC) ranked as the sixth most prevalent cancer and the third most fatal tumour‐related ailment globally. Hepatocellular carcinoma (HCC) constitutes the majority, accounting for 75%–85% of primary liver cancers.^1^ HCC poses a challenge for early detection due to its asymptomatic nature in the initial stages, often resulting in diagnosis at an advanced stage with multiple foci or metastases. Clinical presentation typically occurs when the disease has progressed significantly, with less than 15% of patients undergoing surgical. Advancements in understanding the biological mechanisms underlying HCC could enhance early detection rates, thereby improving patient prognosis.

First identified in the mitochondria of bovine liver in 1953, Glycine N‐acyltransferase (GLYAT) primarily participates in glycine metabolism and various metabolic reactions.^2^ Predominantly found in the human liver and kidney, GLYAT encompasses GLYATL1 and GLYATL2 isoforms. GLYAT orchestrates a crucial biochemical reaction, which catalyses the transfer of an acyl group from an acyl‐CoA to the amino group of glycine, forming an acylglycine and CoASH.^3^ Acyl‐CoA participates in almost all anabolic and catabolic reactions, and results in toxic reaction by depletion of CoASH and accumulated acyl‐CoA itself.^3^ The glucose results in the formation of pyruvate, which requires CoASH in order to be converted to acetyl‐CoA. Therefore, its activity influences mitochondrial adenosine triphosphate (ATP) production, glycine utilization and the toxicity of organic acids, thereby impacting liver metabolism, musculoskeletal development and mitochondrial energy metabolism.^4^ Studies have linked GLYAT dysregulation to tumour growth and metastasis in breast cancer via activation of the PI3K/AKT/Snail pathway.^5^ A bioinformatic‐based screening also underscores the potential of GLYAT as a diagnostic and prognostic marker in kidney renal clear cell carcinoma.^6^ GLYATL1 is overexpressed in prostate cancer^7^ but downregulated in HCC and kidney cancer, with significant prognostic implications for HCC patients.^8^ Despite lower expression levels observed in HCC compared to normal liver tissue,^9^ the precise mechanisms underlying GLYAT's regulation of HCC malignancy and its prognostic relevance remain unclear.

In this study, we elucidated the involvement of GLYAT in HCC development, characterized its underlying mechanisms, and investigated its correlation with patient prognosis and treatment efficacy. Our findings aim to provide valuable insights for early diagnosis, prognosis assessment, and treatment decision‐making in HCC patients.

## 2 MATERIALS AND METHODS

### 2.1 Data collection and clinical samples

The original gene expression data, corresponding prognostic and clinical characteristic data of HCC patients were obtained from the Cancer Genome Atlas (TCGA), the International Cancer Genome Consortium (ICGC) and Gene Expression Omnibus (GEO) datasets. After excluding those without the complete clinical information and intact survival time, 235 patients from TCGA receiving hepatectomy were finally enrolled in this study and were used for further research. All the raw count data were analysed by the package edge R, and the identification of differential expression of GLYAT in HCC tissues and adjacent non‐tumour tissues was also conducted by this package. |log2 (fold change) | > 1 and adjusted *p* value <0.05 were defined as the cut‐off criteria. Seven paired fresh HCC and adjacent non‐tumour tissues were randomized collected in our hospital during surgical resection and stored at −80°C for real‐time quantitative polymerase chain reaction (RT‐qPCR) and Western blotting.

### 2.2 Cell lines

The immortalized liver normal cell line (LO2) and HCC cell lines (Huh 7, HepG2, PLC and SK‐hep1) were donated by the Department of Hepatology Laboratory, Nantong Third Hospital Affiliated to Nantong University. The cell lines mentioned above were cultured in Dulbecco's Modified Eagle's Medium (DMEM, Gibco, USA) or RPMI 1640 medium (Gibco, USA), supplemented with 10% fetal bovine serum (FBS, Gibco, USA) and 1% penicillin and streptomycin (Servicebio, China) in a 5% CO_2_ humidified chamber at 37°C.

### 2.3 RNA interference and lentivirus transduction

In order to overexpress or knockdown GLYAT in HCC cells stably, recombinant lentivirus with green fluorescent protein (GFP) carrying a human GLYAT overexpression plasmid, short hairpin RNA (shRNA) or the corresponding empty control vectors (Vigen, China) were used. The following shRNA target sequence was used: shGLYAT‐b (5′‐GGAAACAGCATTTACAGATTC‐3′). Target cells were cultured at 2 × 10^5^ cells/well in 6‐well plates and infected with lentivirus for 24 h according to the instructions of the manufacturer, followed by selection with 2 μg/mL of puromycin after 48 h. Western blotting analysis and RT‐qPCR were performed to verify the efficiency of overexpression and knockdown. The stable cell lines were designated as Huh 7‐ctrl, Huh 7‐sh, HepG2‐ctrl and HepG2‐oe.

### 2.4 Cell proliferation assays

The ability of cells for proliferation was examined by plate colony formation assays. Cells were plated in 6‐well plates at a concentration of 1000 cells/well and incubated for 14 days in a 5% CO_2_ humidified chamber at 37°C.

### 2.5 Cell migration and invasion assays

For cell invasion assay, transwell chamber containing an 8 μm pore filter membrane (Corning Costar, Corning, USA) with Matrigel (BD Biosciences, Franklin Lakes, USA) was used. 1 × 10^5^ cells were resuspended in 200 μL DMEM without serum into 24‐well upper transwell chamber, and the lower part of the chamber was filled with 800 μL DMEM containing 10% FBS. Whereas for cell migration assay, the Matrigel was omitted. After 24 h culture in a 5% CO_2_ humidified chamber at 37°C, the cells in the upper chamber were removed using a cotton swab, and the cells on the lower surface of the transwell chamber were fixed with 4% paraformaldehyde and stained with 0.1% crystal violet (Beyotime, China) at 37°C for 1 h. Five randomly selected microscopic fields were observed under an Olympus microscope (Olympus, Japan).

### 2.6 Wound healing assay

The stable cell lines were washed with PBS, and 1000 cells were resuspended with DMEM without FBS. Cells were seeded in a 6‐well plate, and then a 200 μL pipette tip was performed to create an artificial straight wound. After 48 h culture, cell migration was observed using an Olympus microscope, and the area between the edges of the wound was calculated by ImageJ 1.53.

### 2.7 Immunohistochemical staining (IHC)

The tissue sections were deparaffinized with xylene, rehydrated with gradient alcohol, antigen retrieved with Sodium Citrate Antigen Retrieval Solution (Solarbio, China) by water bath pot heating, washed with PBS, incubated with 0.3% hydrogen peroxide for 30 min, and then blocked with goat serum for 30 min, blocking endogenous peroxidase activity and nonspecific staining. The tissue sections were incubated with antibody against GLYAT (1:100, Sigma, USA) in a humidified chamber at 4°C overnight. After washing three times with PBS, Goat Anti‐Rabbit IgG H&L (HRP) (1:1000, Abcam, England) antibody was added and incubated for 2 h at room temperature. After three more washes, the 3, 3′‐diaminobenzidine (DAB) (ZSGB, China) and haematoxylin (Solarbio, China) was used to stained. Finally, the sections were successively dehydrated, cleared, sealed and captured.

### 2.8 Western blotting

Cell and tissue proteins were extracted using RIPA buffer (high) (Solarbio, China) and then centrifuged at 12000 r/min for 15 min at 4°C. Protein concentrations were determined using an onedrop protein assay (Thermo Fisher Scientific, USA). After quantification of protein, thirty micrograms of each sample were loaded in the corresponding well and separated on 10% SDS‐PAGE, followed by transferring to polyvinylidene difluoride (PVDF) membranes (Sigma, USA). The membranes were blocked with 5% non‐fat milk for 2 h at room temperature. After washing three times with TBST, the membranes incubated with primary anti‐rabbit antibody of GLYAT (1:1000, Sigma, USA) and β‐actin (1:5000, Abcam, England) overnight at 4°C. Blots were washed again, followed by incubation with the horseradish peroxidase‐conjugated anti‐rabbit secondary antibody (1:10000, Abcam, England) for 2 h. The immunoreactive bands were visualized by the enhanced chemiluminescence system, and the signals were detected by chemiluminescence imager (Tanon, China).

### 2.9 Real‐time quantitative polymerase chain reaction

Total RNA was extracted using the Trizol reagent (Invitrogen, USA) according to the manufacturer's protocol. The obtained RNA (1 μg) was performed to reverse transcribed to cDNA using the HiScript® III RT SuperMix for qPCR (+gDNA wiper) kit (Invitrogen, California, USA). Quantitative real‐time PCR was performed to measure gene mRNA expression using a Taq Pro Universal SYBR qPCR Master Mix kit (Invitrogen, California, USA) on the Roche Real‐time PCR system. The primer sequences for GLYAT were 5′‐3′ (forward) AGACTAGCTTTTCAGGCTAAGGT and 3′‐5′ (reverse) TAAGGATGCTGGGAGGCTCT. The primer sequences for β‐actin were 5′‐3′ (forward) CGCCGCCAGCTCACC and 3′‐5′ (reverse) CACGATGGAGGGGAAGACG. The relative expression of GLYAT was calculated and normalized using the $2^{-\Delta \Delta C_{t}}$ method.

### 2.10 Establish and evaluate a nomogram

Univariate and multivariate Cox regression analysis in terms of OS were performed to investigate the independent prognostic role of the risk model. Based on the results mentioned above, we constructed a nomogram by employing the R package ‘rms’. The accuracy of the established nomogram was effectively measured by evaluation of the calibration curve.

### 2.11 Immune landscape analysis

The ESTMIATE algorithm was applied to evaluate the immune score, stromal score and estimate score based on the proportion of immune and stromal cells in tumour tissues. The CIBEROSRT algorithm was used to calculate the immune infiltration landscape and immune cells in HCC samples.

### 2.12 Immunotherapy efficacy and immunophenoscore

The immunophenoscore (IPS) observed from the Cancer Immunome Atlas (TCIA) database was used to predict the immunotherapy response (anti‐PD‐1 and anti‐CTLA4) between the high and low‐expression groups. The relationship between GLYAT expression and immune checkpoint gene might be related to the therapeutic effect of immune checkpoint inhibitors (ICIs) on tumours. Therefore, we revealed the correlations between GLYAT and immune checkpoints.

### 2.13 Statistical analysis

GLYAT expression between different tissues was assessed using the Student's *t* test. Pearson's Chi‐square test was used to analyse the correlation between *GLYAT* expression and the clinicopathological parameters. Survival analysis was performed through ‘survminer’ package in R. Univariate and multivariate Cox regression analysis were performed to identify the independent indicators related to OS. All statistical analyses were processed using SPSS (Version 23.0), GraphPad Prism (Version 9.5.0) and R programming language (Version 4.1.0) on R Studio. A *p* value <0.05 was considered to be statistically significant.

## 3 RESULTS

### 3.1 GLYAT was downregulated in tumour tissues compared with normal liver tissues

**FIGURE 1:**
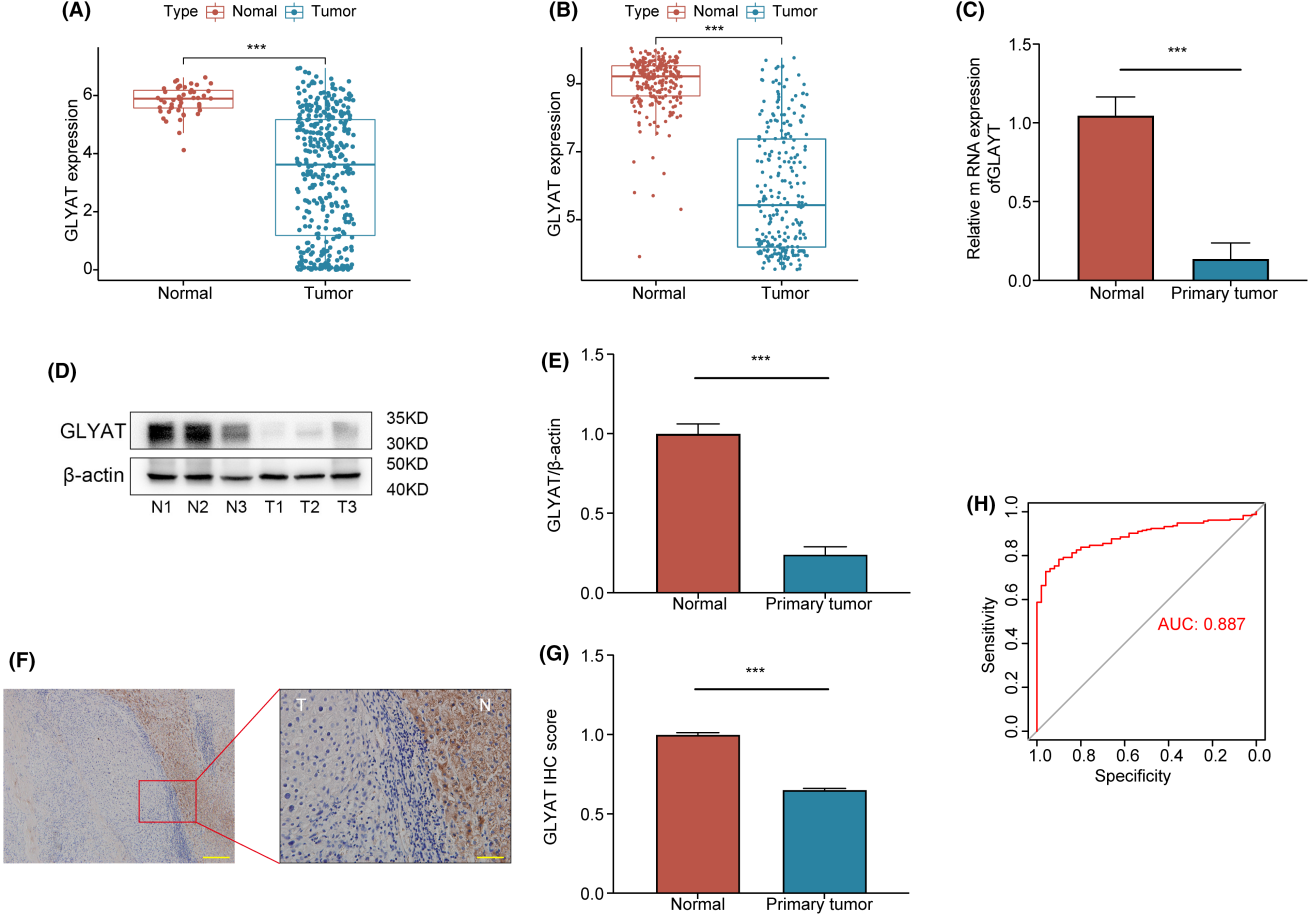
Expression of GLYAT and its diagnostic value in HCC patients. (A, B) The mRNA expression of GLYAT in HCC and paired normal tissues was analysed based on the TCGA and GEO databases. (C–G) RT‐qPCR, Western blotting and IHC were used to analyse the GLYAT expression in fresh HCC tissues and corresponding normal tissues. T, tumour; N, normal. (H) Diagnostic value of GLYAT downregulation for HCC using ROC curve. ***p < 0.001. Scale bars for F are 200 and 50 μm.

In our analysis of mRNA expression levels, we observed a significant downregulation of GLYAT in HCC tissues compared to normal liver tissues in both TCGA and GSE14520 datasets (*p* < 0.0001, Figure 1A,B). This finding was further validated by RT‐qPCR (*p* < 0.0001 Figure 1C), Western blotting (*p* < 0.0001 Figure 1D,E) and immunohistochemistry (IHC) analyses (*p* < 0.0001 Figure 1F,G), which consistently demonstrated lower GLYAT expression in fresh HCC tissues compared to corresponding normal tissues. ROC curve analysis confirmed the diagnostic value of GLYAT in HCC, with an AUC of 0.887 in the TCGA dataset (Figure 1H). These results collectively underscore the potential of GLYAT as a diagnostic marker for HCC.

### 3.2 Low expression of GLYAT correlated with tumour progression in HCC

**FIGURE 2:**
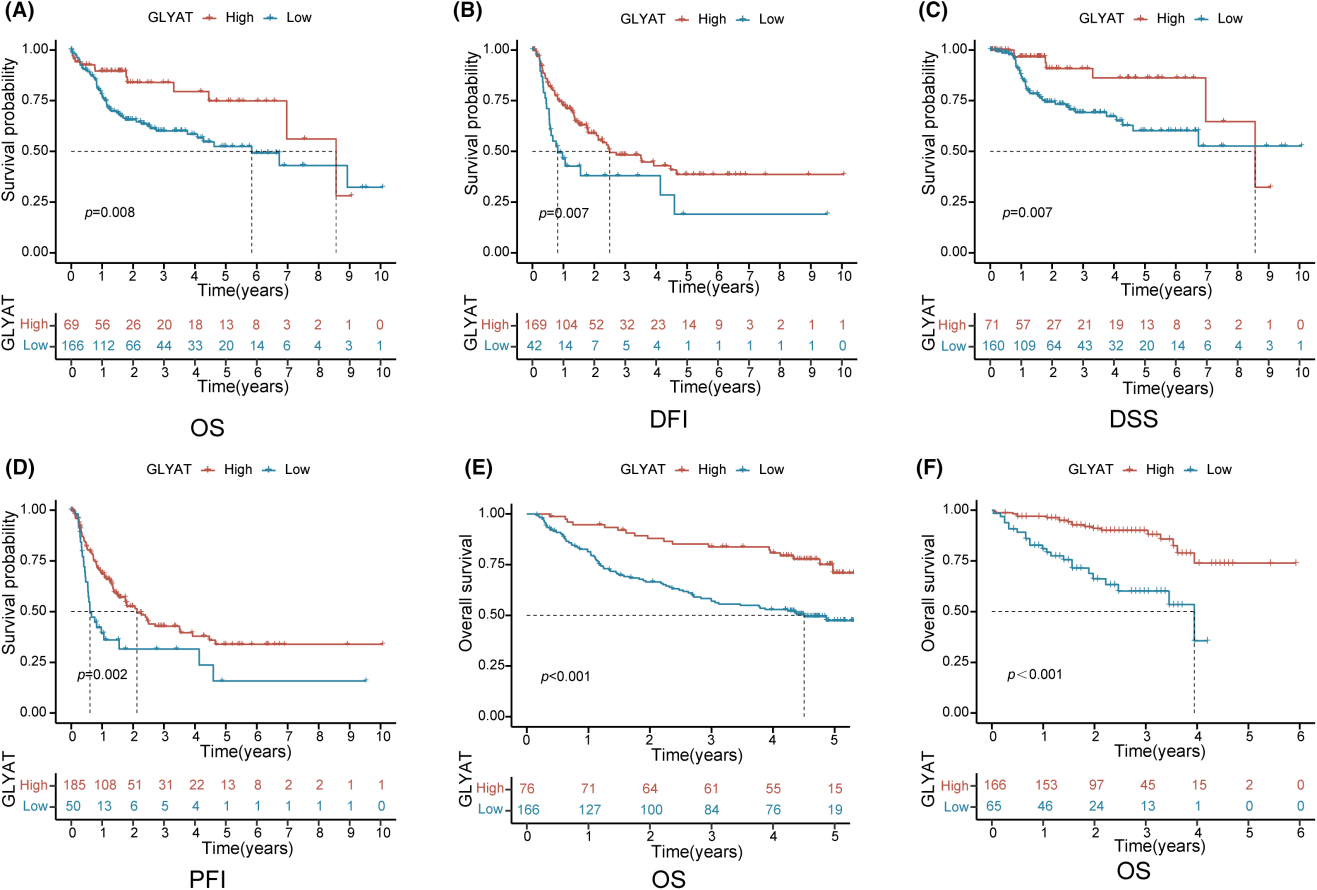
The deficiency of GLYAT predicts poor prognosis in HCC. (A–D) Kaplan–Meier analysis of OS, DFI, DSS and PFI according to GLYAT expression in TCGA database. (E, F) Kaplan–Meier analysis of OS in GEO and ICGC databases.

The prognostic significance of GLYAT in HCC was assessed using the ‘survminer’ package with optimal thresholds. Analysis of the TCGA dataset revealed that HCC patients with low GLYAT expression had poorer OS (*p* = 0.008, Figure 2A), disease‐free interval (DFI) (*p* = 0.007, Figure 2B), disease specific survival (DSS) (*p* = 0.007, Figure 2C) and progression‐free interval (PFI) (*p* = 0.002, Figure 2D). Consistent findings were observed in the GSE14520 and ICGC datasets, where lower GLYAT expression was associated with poorer OS (*p* < 0.001, Figure 2E,F). Clinical analysis of 235 HCC patients from the TCGA database revealed significant associations between GLYAT downregulation and gender (*p* = 0.007) and histologic grade (*p* < 0.001), as shown in Table 1. Additionally, GLYAT expression levels differed significantly among various clinicopathological groups when analysed as a continuous variable (Figure S1A–G). Univariate analysis identified GLYAT expression (*p* = 0.025), TNM stage (*p* < 0.001), T stage (*p* < 0.001) and M stage (*p* = 0.021) as factors correlated with OS (Table 2). Subsequent multivariate Cox regression analysis identified low GLYAT expression (*p* = 0.010) and T3‐T4 stage (*p* = 0.003) as independent prognostic factors affecting patient outcomes (Table 2).

**TABLE 1:** Association between GLYAT expression and the clinical parameters in patients with hepatocellular carcinoma in TCGA.

Clinicopathological characteristics	No. (*n* = 235)	GLYAT expression		*χ* ^2^	*p*
		Low (*n* = 166)	High (*n* = 69)		
Age (years)					
<60	125	95	30	3.701	0.054
≥60	110	71	39		
Gender					
Male	161	105	56	**7.244**	**0.007**
Female	74	61	13		
Histologic grade					
G1	29	14	15	**18.824**	**0.000**
G2	103	66	37		
G3	93	79	14		
G4	10	7	3		
TNM stage					
I	113	73	40	6.641	0.084
II	50	38	12		
III	67	50	17		
IV	5	5	0		
T					
T1–T2	167	115	52	0.878	0.349
T3–T4	68	51	17		
N					
N0	231	162	69	0.558	0.455
N1	4	4	0		
M					
M0	231	162	69	0.558	0.455
M1	4	4	0		

*Note*: Statistically significant *p* values are given in bold, *p* < 0.05.

Abbreviations: GLYAT, Glycine N‐acyltransferase; TNM, tumour‐node‐metastasis.

**TABLE 2:** Cox analysis of overall survival.

Variable	Univariate analysis		Multivariate analysis	
	HR (95% CI)	*p* Value	HR (95% CI)	*p* Value
GLYAT expression				
Low (*n* = 166) vs. high (*n* = 69)	1.943 (1.086–3.476)	**0.025**	2.184 (1.205–3.959)	**0.010**
Age				
<60 (*n* = 125) vs. ≥60 (*n* = 110)	1.201 (0.762–1.891)	0.43		
Gender				
Male (*n* = 161) vs. Female (*n* = 74)	0.778 (0.487–1.244)	0.294		
Histologic grade				
G1–G2 (*n* = 132) vs. G3–G4 (*n* = 103)	1.071 (0.678–1.690)	0.769		
TNM stage				
I (*n* = 113) vs. II–IV (*n* = 122)	1.276 (1.128–1.443)	**<0.001**	1.103 (0.937–1.299)	0.239
T stage				
T1–T2 (*n* = 167) vs. T3–T4 (*n* = 68)	3.103 (1.967–4.896)	**<0.001**	2.561 (1.379–4.756)	**0.003**
N stage				
N0 (*n* = 231) vs. N1 (*n* = 4)	2.064 (0.504–8.448)	0.313		
M stage				
M0 (*n* = 231) vs. M1 (*n* = 4)	3.925 (1.230–12.522)	**0.021**	1.505 (0.456–4.967)	0.502

*Note*: Statistically significant *p* values are given in bold, *P* < 0.05.

Abbreviations: CI, confidence interval; GLYAT, Glycine N‐acyltransferase; HR, hazard ratio; TNM, tumour‐node‐metastasis.

### 3.3 Validation of the prognostic value of GLYAT in HCC based on nomograms

**FIGURE 3:**
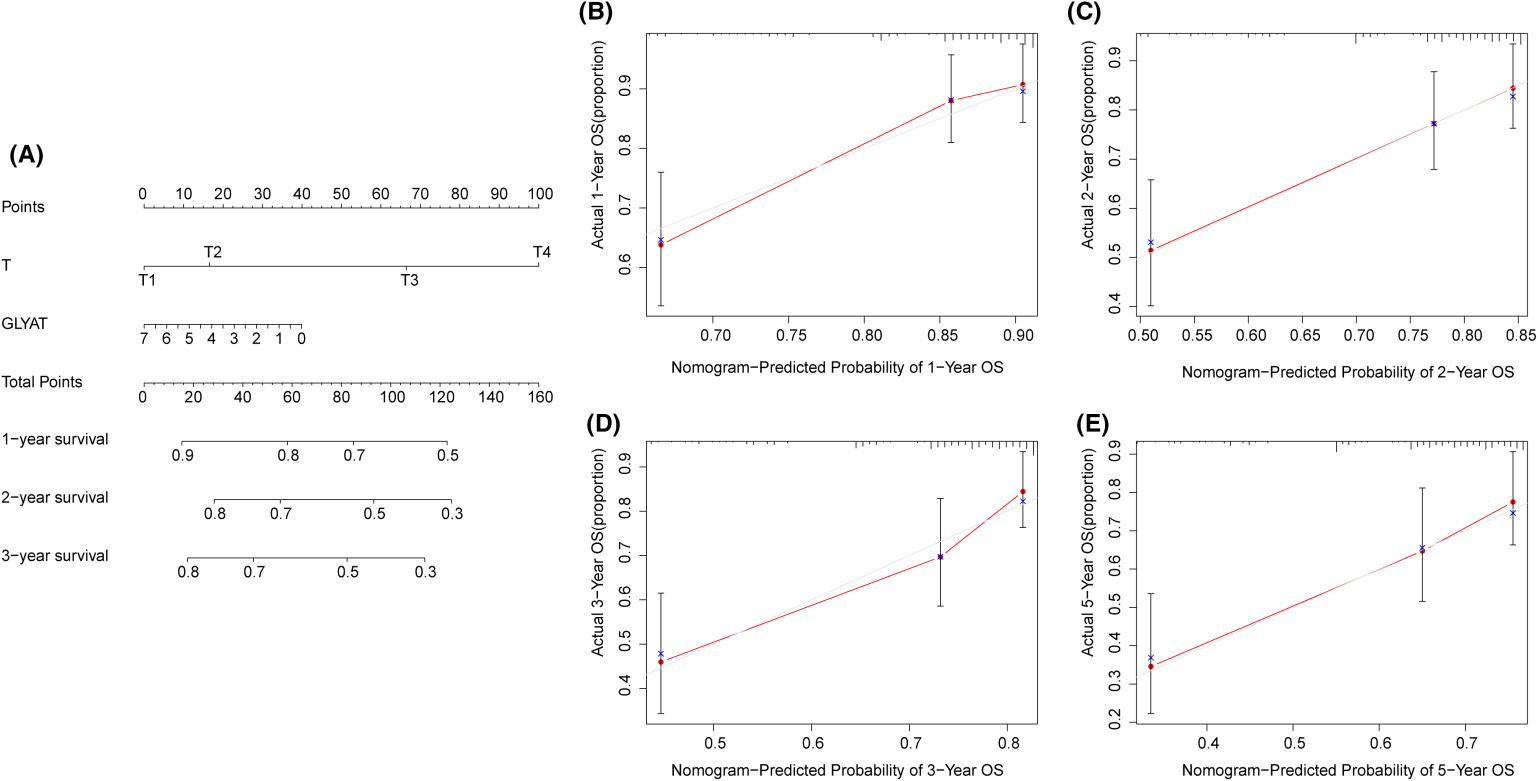
Validation of the prognostic value of GLYAT in HCC. (A) Nomogram predicting the probability of HCC patients' mortality based on three prognostic signatures. (B–E) Calibration curves of the nomogram.

To underscore the prognostic significance of GLYAT in HCC, we developed nomograms incorporating GLYAT mRNA expression and T stage, identified as independent prognostic factors for OS via multivariate analysis. These nomograms were designed to predict the likelihood of survival at 1‐year, 2‐year, 3‐year and 5‐year intervals. Calibration curves demonstrated good agreement between predicted and observed survival rates (Figure 3), further validating the predictive utility of the nomograms.

### 3.4 Down‐regulation of GLYAT strengthened HCC cells proliferation, invasion and migration

**FIGURE 4:**
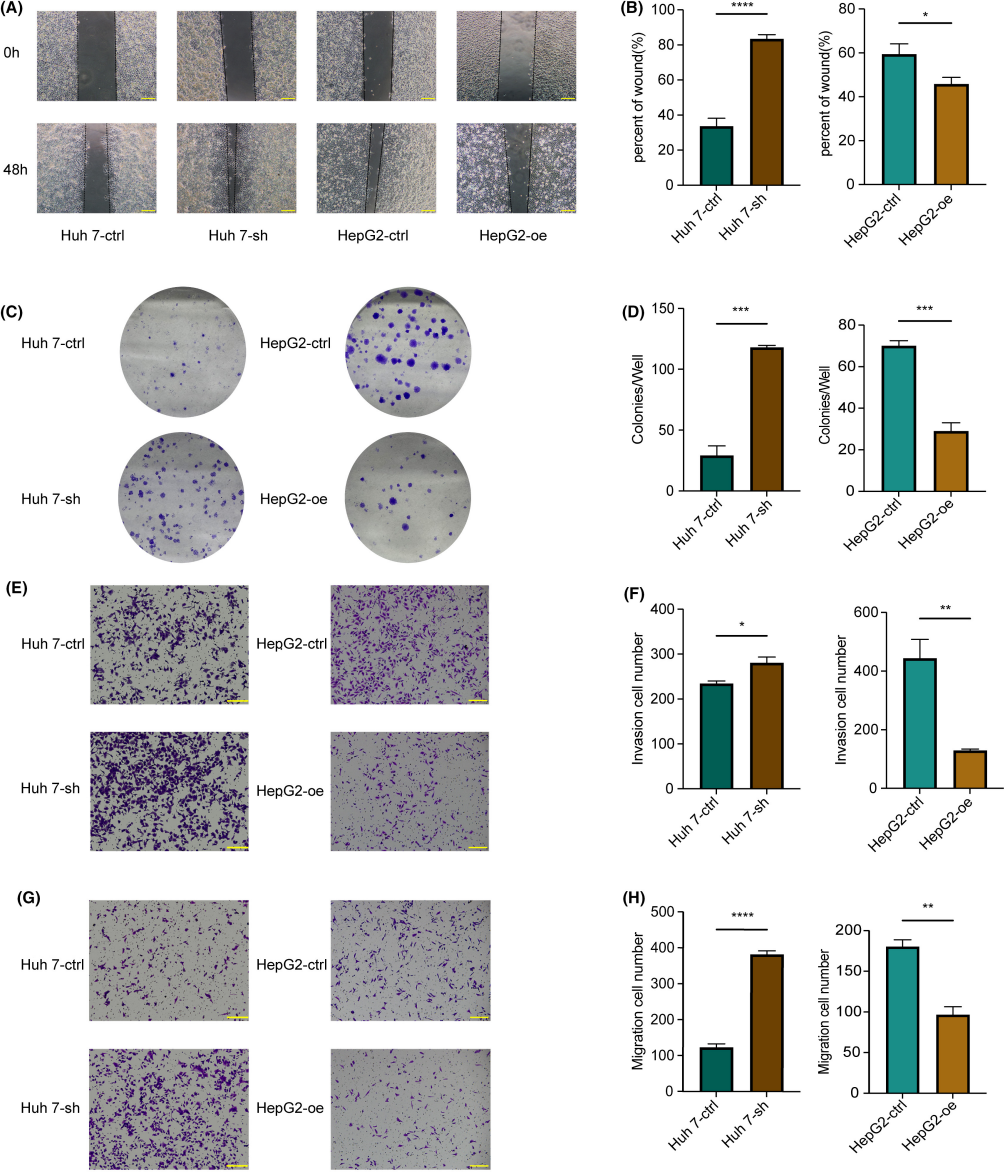
GLYAT suppresses HCC cells proliferation and metastasis in vitro. (A, B) The migration and invasion abilities of Huh 7‐ctrl, Huh 7‐sh and HepG2‐ctrl, HepG2‐oe detected by wound healing assays. (C, D) The proliferation abilities of Huh 7‐ctrl, Huh 7‐sh and HepG2‐ctrl, HepG2‐oe detected by colony formation assays. (E–H) The migration and invasion abilities of Huh 7‐ctrl, Huh 7‐sh and HepG2‐ctrl, HepG2‐oe detected by invasion and migration assays. *p < 0.05; **p < 0.01; ***p < 0.001; ****p < 0.0001. Scale bars for A, E, G are 200 μm.

To further investigate the role of GLYAT in HCC development, we assessed its protein expression levels via Western blotting in the normal liver cell line LO2 and four HCC cell lines (Huh 7, HepG2, PLC and SK‐hep1). We observed lower GLYAT expression in HCC cell lines compared to LO2 cells (Figure S2A,B). Subsequently, we established GLYAT‐overexpression and GLYAT‐knockdown models in HepG2 and Huh 7 cell lines, respectively, which exhibit relatively low and high GLYAT expression. RT‐qPCR (Figure S2C,D) and Western blotting (Figure S2E–H) confirmed the successful construction of these models. We then conducted cell proliferation, wound healing, migration and invasion assays to assess the impact of GLYAT on HCC cell capabilities in vitro. Our findings revealed that downregulation of GLYAT significantly promoted proliferation, invasion and migration of Huh 7 cells. Conversely, HepG2 cells transfected with GLYAT overexpression lentivirus exhibited marked suppression of proliferation, invasion and migration compared to control‐transfected cells (Figure 4A–D).

### 3.5 Correlation between GLYAT expression and tumour immune microenvironment landscape

**FIGURE 5:**
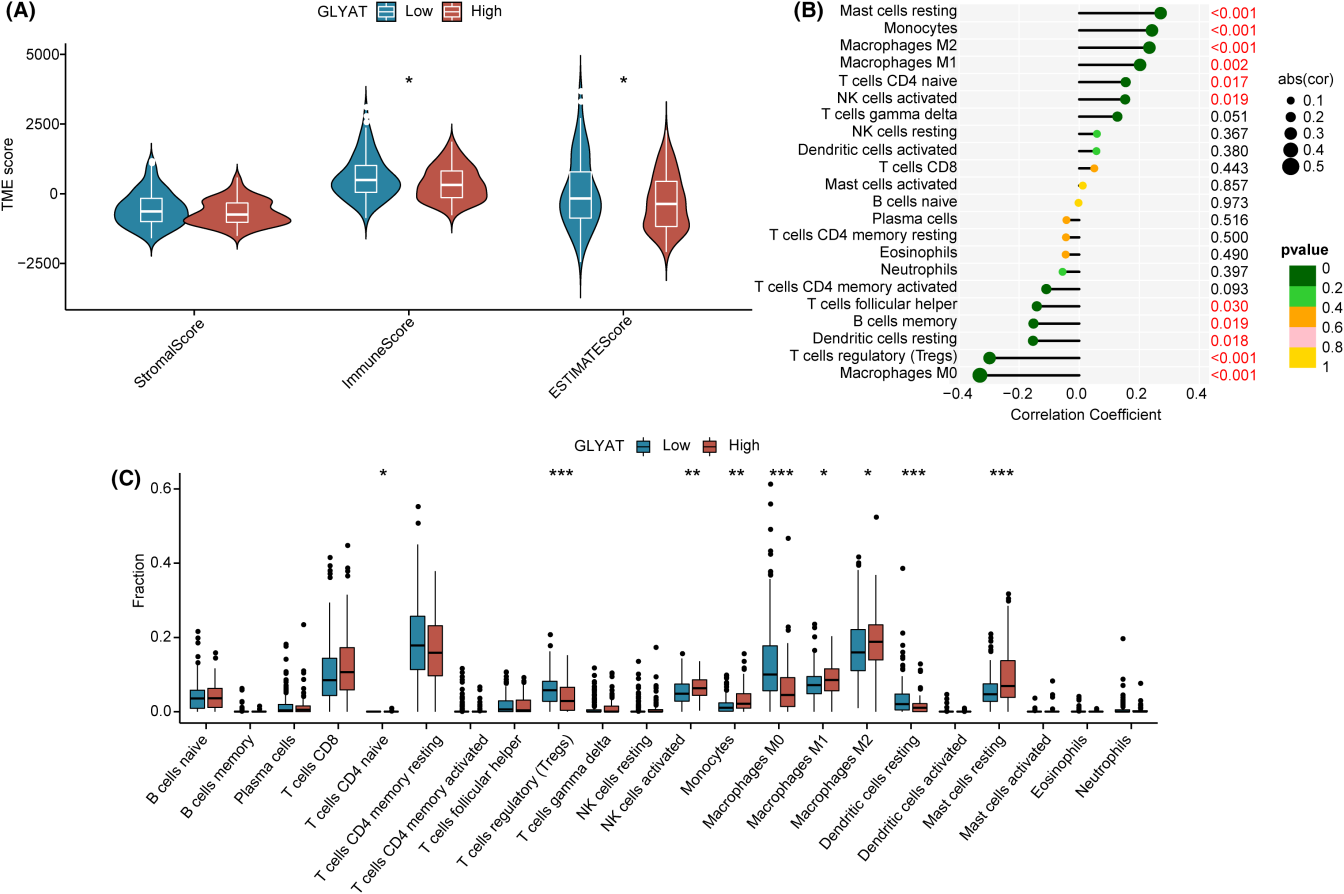
The landscape of tumour immune microenvironment. (A) StromalScore, ImmuneScore and ESTIMATEScore between high‐ and low‐GLYAT group in TCGA. (B) Correlation between GLYAT expression and immune cells in TCGA. (C) Relative cell abundance immune cells by CIBERSORT between two groups in TCGA. *p < 0.05; **p < 0.01; ***p < 0.001.

Tumour‐infiltrating immune cells play a crucial role in cancer prognosis, including HCC. We employed two methods, CIBERSORT and ESTIMATE, to assess immune cell infiltration and explore the interaction between GLYAT expression and the tumour immune microenvironment. According to ESTIMATE analysis, GLYAT expression showed no significant correlation with stromal score (*p* > 0.05). However, the immune score and ESTIMATE score were higher in the low GLYAT expression group compared to the high GLYAT expression group (*p* < 0.05, Figure 5A). Subsequently, using the CIBERSORT algorithm, we found positive correlations between GLYAT expression and mast cells resting, monocytes, macrophages M2, macrophages M1, T cell CD4 naive and NK cells activated in the tumour immune microenvironment. Conversely, GLYAT expression showed negative correlations with T cell follicular helper, B cell memory, Dendritic cell resting, regulatory T cells (Tregs) and Macrophages M0 (Figure 5B). Additionally, patients with low GLYAT expression exhibited increased proportions of Tregs, Macrophages M0 and dendritic cell resting, while those with high GLYAT expression showed elevated levels of T cell CD4 naive, NK cells activated, monocytes, macrophages M1, macrophages M2 and mast cells resting (Figure 5C). These findings underscore the association of GLYAT expression with the tumour immune microenvironment and its potential as an indicator of immune status in HCC patients.

### 3.6 GLYAT expression was associated with immunotherapy response and immune checkpoints

**FIGURE 6:**
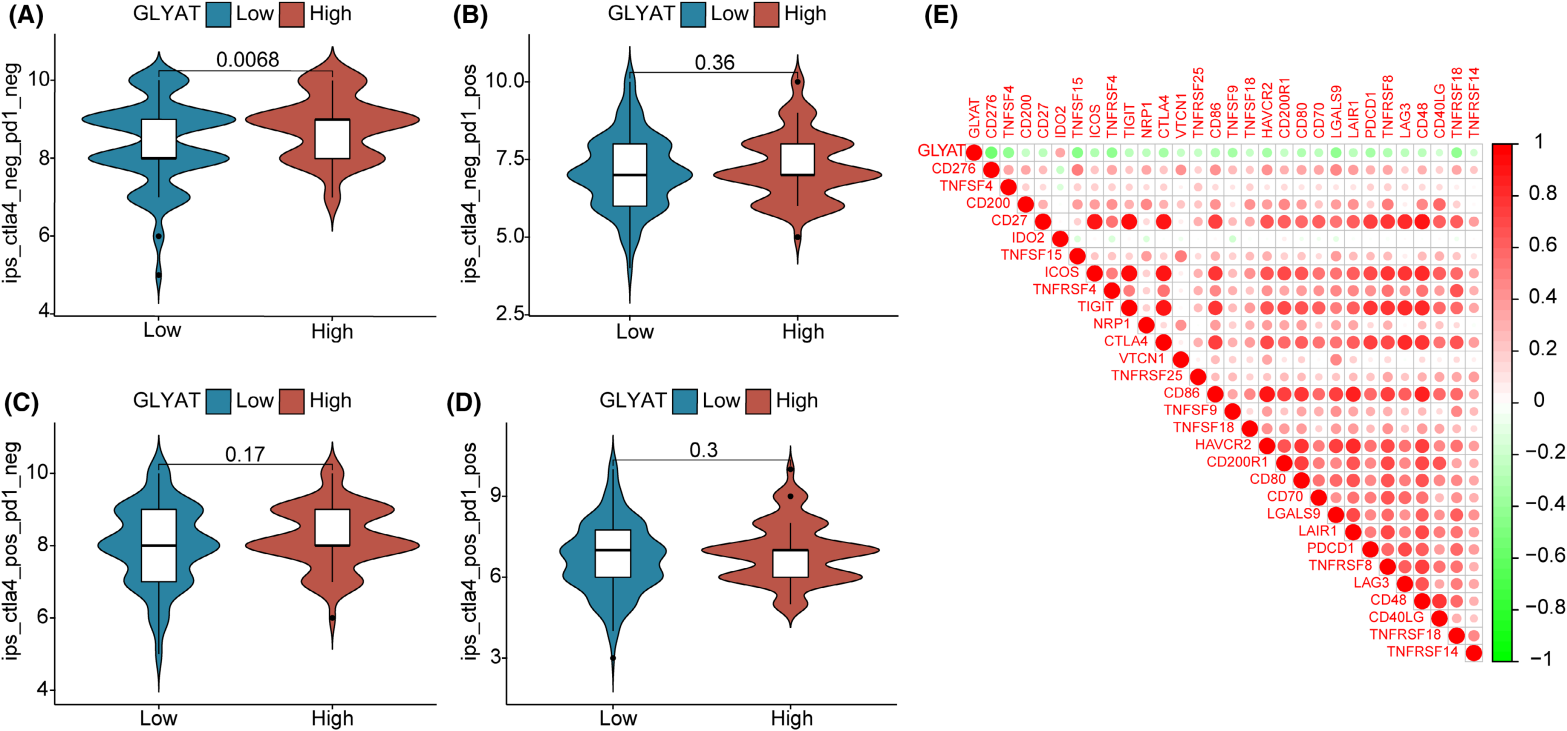
The estimation of Immunotherapy response. (A–D) The association between high‐ and low‐GLYAT group and immunophenoscore (IPS). (E) The correlation between the expression of immune checkpoint genes and GLYAT expression.

Considering the distinct tumour immune microenvironment in groups with low and high GLYAT expression, we conducted IPS analysis to assess the potential responsiveness to immunotherapy targeting PD‐1 and CTLA‐4. The findings suggested that ICIs could potentially offer greater efficacy in patients with low GLYAT expression (Figure 6A). Furthermore, we investigated the correlation between GLYAT expression and immune checkpoint genes. Interestingly, GLYAT expression exhibited a negative correlation with most immune checkpoint genes, except for IDO2 (Figure 6B). These insights suggest that patients with low GLYAT expression may demonstrate enhanced sensitivity to ICIs, offering prospects for tailored precision therapy in HCC patients.

## 4 DISCUSSION

In recent years, HCC remains a significant global health challenge with high morbidity and mortality rates.^1^ Advances in genetics and molecular biology have underscored the importance of identifying key biomarkers for early detection and personalized tumour therapy. Notably, GLYAT has emerged as a consistently downregulated gene across various tumour types, including HCC.^8^, ^9^, ^10^, ^11^ Despite this, the precise biological functions and molecular mechanisms of GLYAT in HCC have remained elusive. Our study revealed that GLYAT expression was significantly reduced in HCC tissues compared to adjacent noncancerous tissues, and its low expression correlated with poorer OS. Correlation, ROC and regression analyses suggested that GLYAT could serve as an independent predictor of survival in HCC patients. Additionally, a nomogram based on GLYAT mRNA expression and T stage exhibited high precision. Furthermore, in vitro functional experiments demonstrated that low GLYAT expression markedly promoted the malignant behaviour of tumour cells. Subsequent investigations revealed associations between GLYAT expression and the tumour immune microenvironment, as well as immunotherapy sensitivity. In summary, GLYAT shows promise as a potential diagnostic and prognostic predictor, as well as a useful marker for predicting tumour immunotherapy responses in HCC patients.

In line with the bioinformatics analysis, our experimental findings from RT‐qPCR, Western blotting and IHC consistently demonstrated downregulation of GLYAT in HCC tissues compared to normal tissues. This pattern was also observed in HCC cells compared to liver cells. Our results align with those of Guan et al.,^8^ where Kaplan–Meier survival analysis indicated that HCC patients with high expression of GLYATL1 had a better prognosis, supporting our findings. Furthermore, our study revealed that GLYAT mRNA expression exhibited excellent diagnostic value for HCC according to ROC curves, suggesting its potential as a biomarker for HCC diagnosis.

The clinical relevance of GLYAT expression in HCC was further investigated, revealing significant correlations with patient age, gender, histological grading and clinical stage. Decreased GLYAT expression was associated with higher histological grade and advanced clinical stage, suggesting a potential role in HCC progression and tumourigenesis. However, some findings seemed contradictory, such as higher GLYAT expression in patients over 65 years of age despite their generally poorer prognosis, and lower expression in women despite the lower incidence of HCC in females.^1^, ^12^, ^13^ Due to the individual variability in the prognosis of HCC patients, a large number of genomic clinicopathological prognostic models have been developed and applied.^14^, ^15^ To address the variability in HCC prognosis, nomograms were constructed based on GLYAT mRNA expression and T stage, identified as independent risk factors for OS. Calibration curves demonstrated good agreement between predicted and actual survival rates, offering a novel approach for prognostic assessment in HCC patients.

Prior research has demonstrated that the downregulation of GLYAT enhances invasion, migration and proliferation of breast cancer cells by modulating epithelial‐mesenchymal transition (EMT), a finding validated in animal models.^5^ Given that the poor prognosis of HCC is often attributed to early tumour metastasis and recurrence, with EMT playing a crucial role in intra‐ and extrahepatic metastasis,^16^ we delved into GLYAT's role in HCC through in vitro experiments. GLYAT‐overexpressing and knockdown cell lines were established based on GLYAT expression levels in hepatocellular cell lines, followed by assays for cell proliferation, invasion and migration. Results revealed that GLYAT overexpression significantly inhibited the proliferation, invasion and migration of hepatocellular cell lines, suggesting a potential role in mitigating tumour recurrence, metastasis and poor prognosis, in line with previous findings.^8^ Hence, GLYAT emerges as a pivotal target gene influencing HCC progression, with increased GLYAT expression potentially attenuating the malignancy of hepatocellular carcinoma. In recent years, ICIs have revolutionized cancer treatment, including HCC, with significant improvements in patient survival. The liver, being a crucial immune organ, harbours a diverse array of immune cells and alterations in the liver's immune microenvironment play a pivotal role in HCC development and regression. The tumour immune microenvironment (TME) has garnered considerable attention due to its association with HCC prognosis. Numerous studies have highlighted the prognostic significance of immune cell infiltration in the TME of HCC patients, prompting an investigation into the relationship between GLYAT expression and immune cell infiltration in HCC.^17^ Our findings revealed a positive correlation between GLYAT expression and resting mast cells, monocytes, M2 macrophages, M1 macrophages, naive CD4+ T cells and activated NK cells. Conversely, GLYAT expression exhibited a negative correlation with helper follicular T cells, memory B cells, resting dendritic cells, Tregs and M0 macrophages. Notably, NK cells play a crucial role in early immune responses and confer protection against liver cancer,^18^ whereas Tregs cells induce immune tolerance and suppress tumour‐specific T cell activity, correlating with poor prognosis in HCC patients.^19^ Our results align with these findings, suggesting that low GLYAT expression may predispose HCC patients to immune escape and poorer outcomes. Additionally, previous studies have demonstrated the efficacy of ICIs in HCC patients, implicating multiple immune checkpoints in immune tolerance and tumour immunosuppression.^20^, ^21^, ^22^, ^23^ Our study further explored the correlation between GLYAT expression and immune checkpoints, revealing a negative association between GLYAT expression and most immune checkpoints. Furthermore, our immunophenotype scoring system indicated that HCC patients with low GLYAT expression may show a better response to ICIs. Collectively, these findings underscore the potential of GLYAT as a valuable target in HCC immunotherapy. Inhibition of GLYAT expression levels may enhance the efficacy of ICIs, offering a promising avenue for HCC immune therapy.

Indeed, our study has certain limitations that warrant acknowledgment. While we briefly investigated the correlation between GLYAT and immunotherapy, the scope of this aspect was relatively limited. Although we validated some findings using clinical samples and explored underlying mechanisms through in vitro experiments, the sample size was modest. To strengthen the robustness of our conclusions, larger cohorts of clinical samples and additional in vivo and ex vivo experiments are necessary. Further studies involving a larger number of cases are essential to elucidate the precise role of GLYAT in HCC comprehensively.

In conclusion, our study confirms that GLYAT expression is downregulated in HCC tissues, and patients with higher GLYAT expression exhibit better prognosis compared to those with lower expression levels. Furthermore, overexpression of GLYAT significantly suppresses the malignant phenotype of HCC cell lines in vitro. Additionally, patients with low GLYAT expression may demonstrate an enhanced response to immunotherapy. Thus, GLYAT holds promise as a potential screening target for early‐stage HCC patients, offering valuable insights into prognosis and treatment strategies, thereby potentially improving clinical outcomes for patients.

## AUTHOR CONTRIBUTIONS

**Fengchen Jiang:** Conceptualization (equal); investigation (equal); methodology (equal); validation (equal); writing – original draft (equal); writing – review and editing (equal). **Shuiping Zhou:** Conceptualization (equal); formal analysis (equal); methodology (equal); visualization (equal); writing – original draft (equal). **Chuanlong Xia:** Formal analysis (equal); investigation (equal); methodology (equal); visualization (equal). **Jiale Lu:** Data curation (lead); investigation (equal); methodology (equal). **Bin Wang:** Investigation (equal); methodology (equal). **Xiaowei Wang:** Funding acquisition (supporting); investigation (equal). **Jiandong Shen:** Writing – review and editing (supporting). **Wei Ding:** Investigation (equal). **Mengjie Yin:** Investigation (equal). **Feng Dai:** Conceptualization (equal); methodology (equal); supervision (equal); writing – review and editing (equal). **Shouzhong Fu:** Conceptualization (equal); funding acquisition (lead); supervision (equal); writing – original draft (equal); writing – review and editing (equal).

## FUNDING INFORMATION

The author(s) declare financial support was received for the research, authorship and/or publication of this article. This work was supported by Nantong Science and Technology Project (MS12021034, MSZ2023020 and JCZ2022024).

## CONFLICT OF INTEREST STATEMENT

The authors confirm that there are no conflicts of interest.

## Supporting information


Figure S1.

## Data Availability

The data that support the findings of this study are available from the corresponding author upon reasonable request.
