# Supplementary material for: Downregulation of GLYAT correlates with tumour progression and poor prognosis in hepatocellular carcinoma
Source: J Cell Mol Med. 2024 Nov 4;28(21):e70197. doi: 10.1111/jcmm.70197 (PMC11534071; doi:10.1111/jcmm.70197)
Supplement: Supplementary file 1 — Figure S1. [file JCMM-28-e70197-s001.docx]

Supplementary Material

# Supplementary Figures and Tables

## Supplementary Figures

**
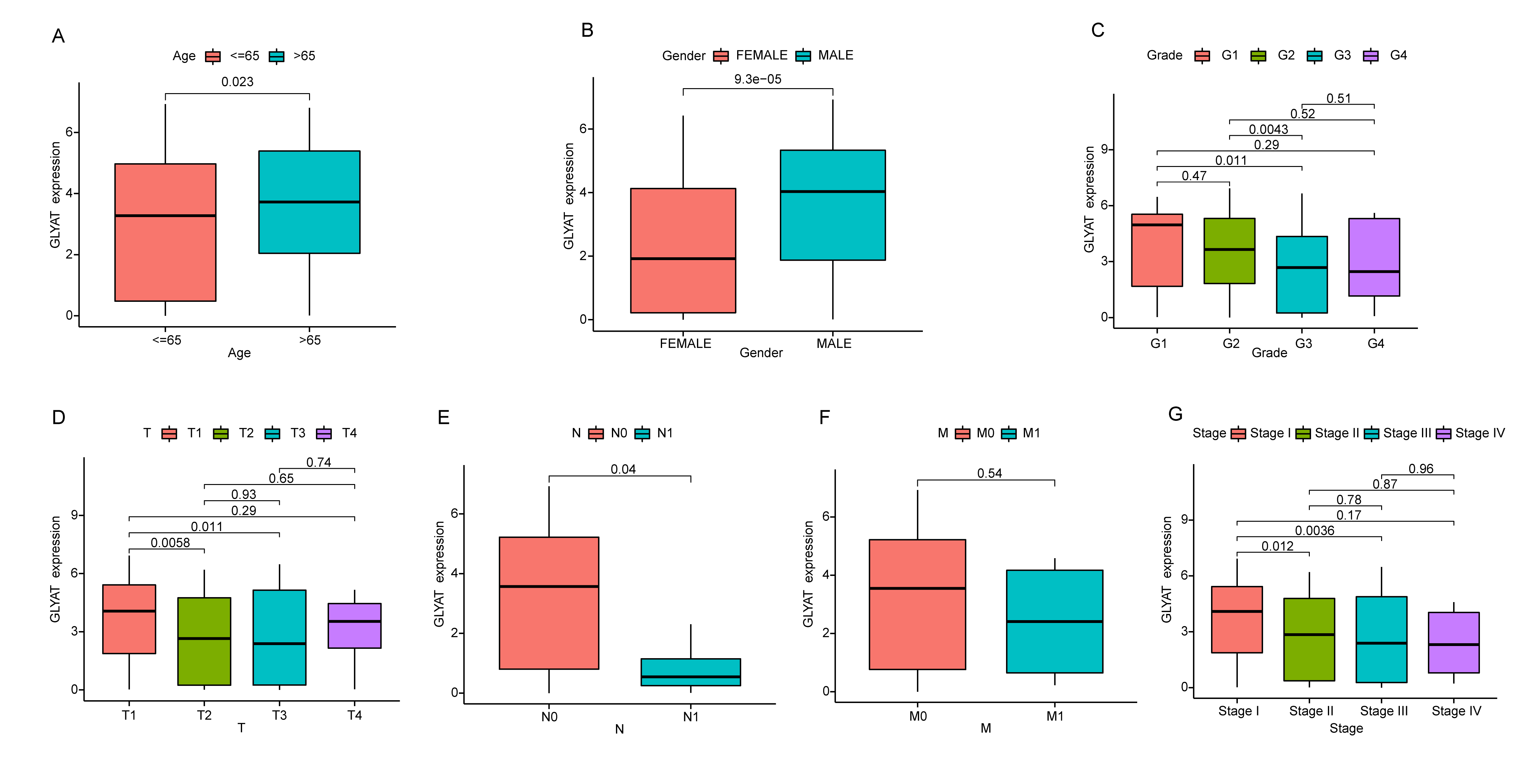
**

**Supplementary Figure 1.** Comparison of GLYAT expression in different clinicopathological groups. (**A-G**) Comparison of GLYAT expression in different groups of age, gender, histologic grade, T stage, N stage, M stage and TNM stage.


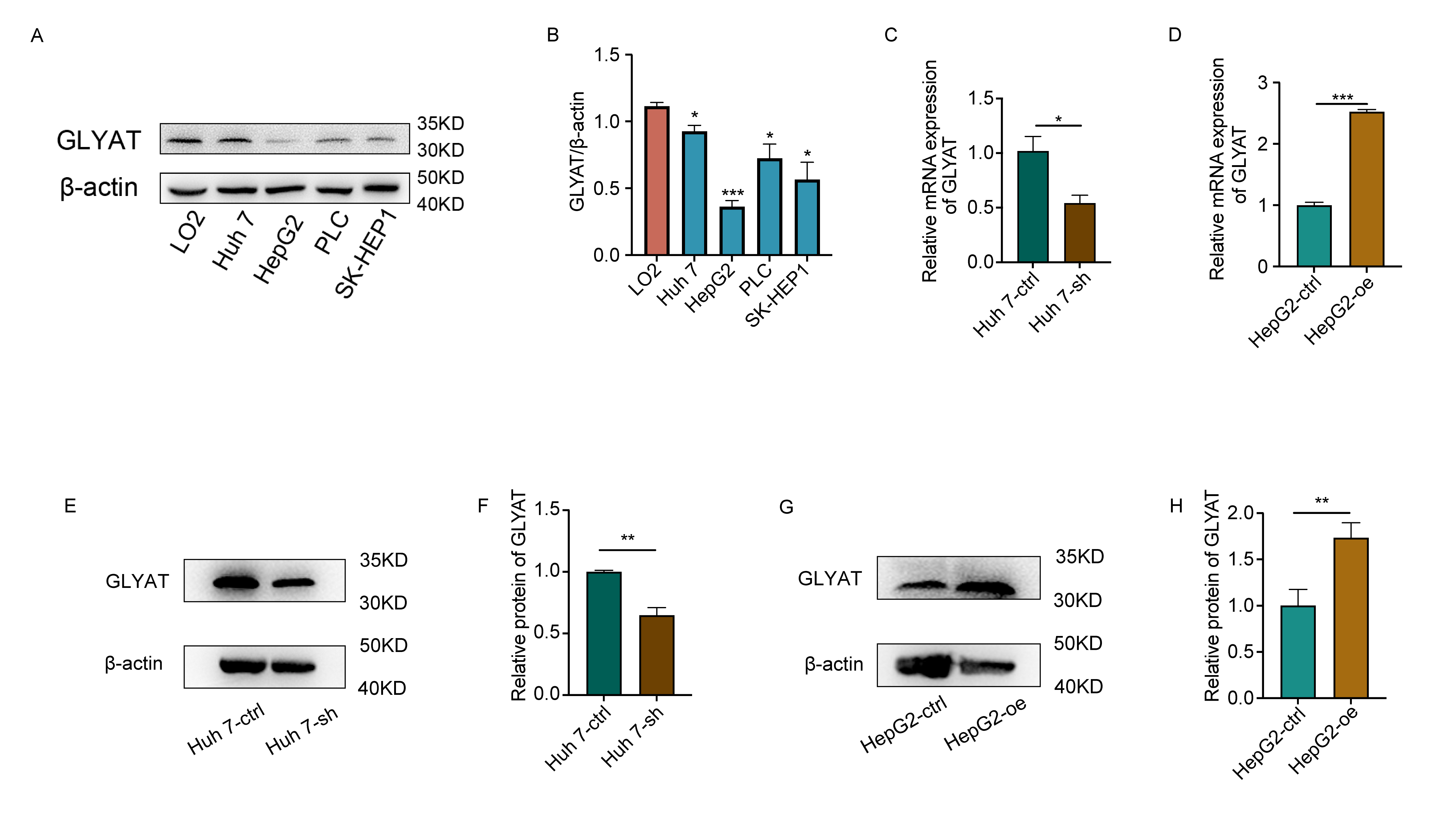


**Supplementary Figure 2.** GLYAT was downregulated in HCC cells. (**A, B**) Western-blot was used to analyze the expression of GLYAT in LO2 normal liver cell line and different HCC cell lines. (**C-G**) Lentivirus infection was performed in Huh 7 and HepG2 cell lines. The efficiency of knockdown in Huh 7 cell line and overexpression of GLYAT in HepG2 cell line were evaluated via RT-qPCR and Western blotting. **P* < 0.05; ***P* < 0.01; ****P* < 0.001.
